# Angioembolization as a life-saving maneuver for unstable pelvic fractures in skeletally immature children: a multicenter case series

**DOI:** 10.3389/fped.2025.1663214

**Published:** 2025-12-04

**Authors:** Hui Li, Guangbin Huang, Yunfeng Yi, Junhua Guo, Yong Luo, Yong Fu, Anyong Yu, Gongliang Du, Mao Zhang, Dingyuan Du

**Affiliations:** 1 Department of Traumatology, Chongqing University Central Hospital, Chongqing Emergency Medical Center, Chongqing, China; 2 Department of Cardiothoracic Surgery, The 909th Hospital, School of Medicine, Xiamen University, Zhangzhou, China; 3 Department of Emergency, The Second Affiliated Hospital, Hengyang Medical School, University of South China, Hengyang, China; 4 Trauma Center, Pediatric Orthopedic Department, The Second Affiliated Hospital, Hengyang Medical School, University of South China, Hengyang, China; 5 Department of Emergency, Affiliated Hospital of Zunyi Medical University, Zunyi, China; 6 Emergency Surgery Department, Shaanxi Provincial People's Hospital, Xi'an, China; 7 Department of Emergency Medicine, Second Affiliated Hospital, Zhejiang University School of Medicine, Hangzhou, Zhejiang, China

**Keywords:** children, adolescent, pelvic fracture, internal iliac artery, angiography, embolization, hemorrhagic shock

## Abstract

**Background:**

Hemodynamically unstable pelvic fractures (HUPF) in skeletally immature children and adolescents carry significant mortality. While internal iliac artery embolization (IIAE) is a cornerstone of management for HUPF in adults, data on its application and outcomes in this truly pediatric population, particularly in China, are scarce. This study aimed to evaluate the feasibility, safety, and clinical outcomes of IIAE for HUPF in this specific, vulnerable group.

**Methods:**

This multicenter, retrospective case series included patients aged 15 years or younger who presented with HUPF and underwent IIAE at three major Chinese trauma centers between 2019 and 2023. Key outcomes included immediate hemorrhage control, in-hospital mortality, and long-term complications.

**Results:**

A total of seven patients underwent IIAE. The cohort demonstrated severe trauma, with a median Pelvic Abbreviated Injury Scale (AIS) of 5 and a median Injury Severity Score (ISS) of 36. Of the six patients who underwent contrast-enhanced computed tomography (CECT), active arterial extravasation was identified in four. However, subsequent angiography confirmed life-threatening arterial injuries in all seven patients, including the two with negative CECT scans. Immediate and sustained hemodynamic stability was achieved in six patients (85.7%). The single mortality occurred in a patient with refractory hemorrhagic shock who required a massive transfusion of 28.5 units of red blood cells, whereas the median for the six survivors was 4 units. At a median follow-up of 22 months, all survivors were ambulatory and no major procedure-related ischemic complications were observed.

**Conclusion:**

IIAE is a feasible and effective life-saving intervention for HUPF in skeletally immature patients. A negative CECT scan does not rule out significant arterial injury, underscoring the vital role of prompt angiography. We recommend a low threshold for early intervention, with consideration of non-selective embolization as a primary damage control tactic in these critically injured children.

## Introduction

Hemodynamically unstable pelvic fractures (HUPF) represent a life-threatening manifestation of severe trauma, posing a unique challenge in skeletally immature children and adolescents. While overall pelvic fractures in this demographic are relatively uncommon, with reported incidences ranging from 0.2% to 7.5% (1), HUPF cases are associated with disproportionately high mortality rates of up to 14% (2, 3). It is in this younger, skeletally immature population that the differences from adult patients are most profound, due to distinct pelvic anatomy, biomechanics, and physiological responses to shock (4). In adult trauma care, internal iliac artery embolization (IIAE) is a cornerstone of hemorrhage control (5). However, robust evidence guiding its use in the truly pediatric population remains scarce, particularly from Chinese trauma centers.

**Figure 1:**
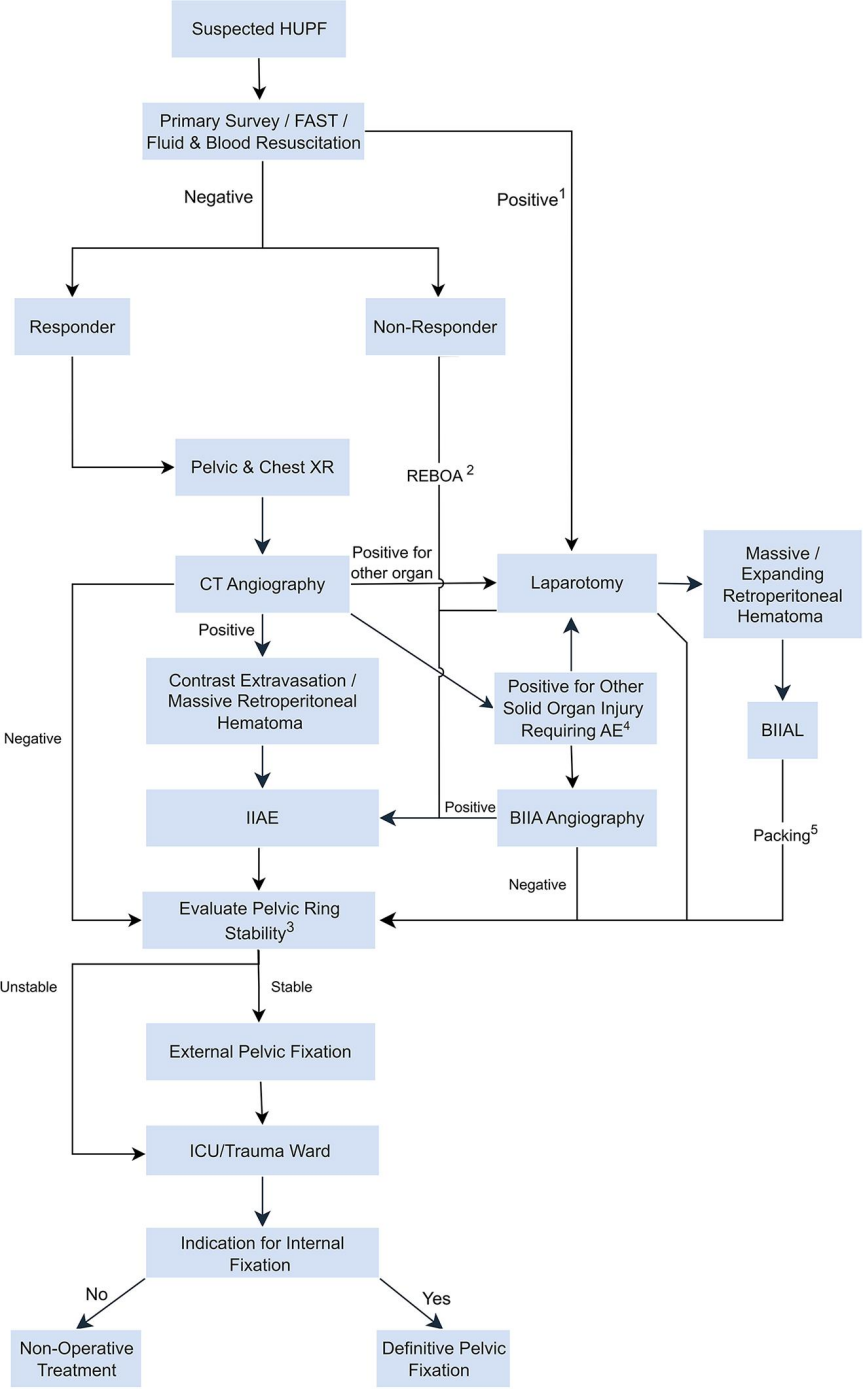
Management algorithm for pediatric hemodynamically unstable pelvic fracture (HUPF). This algorithm represents a general pathway and must be adapted based on clinical judgment and the patient's physiological response. For example, a patient may require repeat imaging and embolization after an initial laparotomy. AE: Arterial Embolization; BIIAL: Bilateral Internal Iliac Artery Ligation; CT: Computed Tomography; FAST: Focused Assessment with Sonography for Trauma; HUPF: Hemodynamically Unstable Pelvic Fracture; ICU: Intensive Care Unit; IIAE: Internal Iliac Artery Embolization; REBOA: Resuscitative Endovascular Balloon Occlusion of the Aorta; XR: x-Ray. 1. While a positive FAST (Focused Assessment with Sonography for Trauma) can indicate thoracoabdominal injury, this workflow primarily addresses cases of significant intra-abdominal hemorrhage. A FAST-positive patient may still proceed to CT angiography if their hemodynamic status allows; 2. REBOA is not a routine procedure but an adjunctive, life-saving measure that serves as a bridge to definitive hemorrhage control; 3. Pelvic ring stability is determined based on the AO-OTA classification. External pelvic fixation is indicated for mechanically unstable fracture patterns (e.g., most Type B and all Type C fractures); 4. If CECT identifies another major source of hemorrhage (e.g., a solid organ injury), a pelvic angiogram should still be strongly considered in the presence of a high-risk pelvic fracture to rule out a concurrent or occult pelvic arterial injury; 5. After bilateral internal iliac artery ligation (BIIAL), pelvic packing for the retroperitoneal hematoma may be performed as part of a damage control laparotomy, which can conclude with temporary abdominal closure.

The management of pediatric HUPF is a race against time where rapid hemorrhage control is the paramount objective. These challenges are particularly acute in skeletally immature patients, whose unique physiology can be deceptive. Their remarkable ability to compensate for significant blood loss often masks the true severity of injury, leading to a state of compensated shock that can rapidly deteriorate without warning (6). This, coupled with concerns about radiation exposure and the long-term effects of intervention on a growing pelvis, contributes to a lack of standardized, proactive treatment protocols for this specific age group (7).

Therefore, this study was designed to address this critical evidence gap. We retrospectively analyzed our multicenter experience with IIAE for HUPF specifically in children and early adolescents (aged 15 years and under). By focusing on this distinct demographic, we aimed to provide targeted clinical evidence to support the development of more robust emergency treatment protocols for this vulnerable and often-overlooked patient group.

## Methods

### Study design and patient population

We performed a retrospective multicenter review of medical records from three major trauma centers in China, spanning from January 2019 to December 2023. This study received approval from the Institutional Review Board (IRB) at each participating center, with patient consent requirements waived.

The study population comprised all patients aged 15 years or younger who underwent IIAE for hemorrhage control in the setting of an acute, traumatic pelvic fracture.

Hemodynamic instability was defined according to pediatric Advanced Trauma Life Support (ATLS) guidelines by the presence of one or more of the following criteria. First, evidence of compensated shock, defined as persistent age-specific tachycardia in conjunction with signs of poor organ perfusion (e.g., prolonged capillary refill time, weak peripheral pulses, or cool, mottled extremities). Second, the presence of age-specific hypotension, defined as a systolic blood pressure (SBP) < 90 mmHg for patients >10 years, or < (70 mmHg + [2 × age in years]) for patients aged 1–10 years. This definition prioritizes the early signs of compensated shock, as the development of hypotension in children indicates a state of severe decompensated shock, often signifying a blood loss of greater than 45%.

### Data collection

A standardized data collection form was used to extract information from each patient's electronic medical record. Key data points included:

Patient and Injury Characteristics: Age, sex, mechanism of injury, Pelvic Abbreviated Injury Scale (AIS), and Injury Severity Score (ISS).

Hemodynamic Status on Admission: This included initial vital signs (systolic blood pressure, heart rate) and the patient's response to initial fluid and blood product resuscitation.

Diagnostic and Procedural Information: Key findings from contrast-enhanced CT (CECT), details of the angiographic procedure and embolization technique (e.g., embolic agents, vessels targeted), and any adjunctive hemostatic measures used (e.g., REBOA, pelvic packing).

Clinical Outcomes: Data on the achievement of hemodynamic stability post-procedure, in-hospital mortality, length of hospital stay, and any procedure-related complications were collected.

Follow-up Data: Available information from outpatient records regarding functional recovery (e.g., ambulatory status) and fracture healing was reviewed.

All collected data were anonymized and analyzed descriptively.

## Results

### Patient characteristics and injury severity

During the study period, a total of seven patients meeting the inclusion criteria were identified from four trauma centers. The cohort comprised three males and four females, with a median age of 14 years (Table 1). The primary mechanism of injury was a high-fall injury in four cases. Three of the seven patients were transferred from local hospitals for higher-level care, which was associated with prolonged pre-hospital times ranging from 420 to 480 min. In contrast, the pre-hospital time was markedly shorter for the four non-transferred patients; except for one case, all were admitted within 30 min of injury. The patients presented with severe polytrauma, evidenced by a median Injury Severity Score (ISS) of 36 (range: 22–50). The pelvic injuries were particularly severe, with a median pelvic Abbreviated Injury Scale (AIS) of 5, and mechanically unstable AO Foundation and Orthopaedic Trauma Association (AO-OTA) Type C fractures were present in five of the seven cases (Table 1).

**Table 1:** Emergency assessment characteristics of seven pediatric and adolescent patients with hemodynamically unstable pelvic fractures.

Patient	Sex	Age	Injury mechanism	Hospital transfer	Prehospital time	Admission SBP/DBP (mmHg)	Admission HR (bpm)	GCS	ISS	Pelvic AIS	AO-OTA classification
A	M	12	HFI	No	240 min	104/65	110	10	33	5	C2.3
B	F	12	HFI	Yes	420 min	75/45	120	12	29	5	C1.3
C	M	12	PTAI	No	27 min	93/55	125	10	38	5	C1.3
D	M	14	HFI	No	30 min	80/53	178	3	38	5	C2.2
E	F	14	PCAI	Yes	420 min	110/56	105	15	36	4	B3.1
F	F	15	HFI	Yes	480 min	89/75	145	12	50	5	C1.3
G	F	15	PTAI	No	24 min	108/74	102	13	22	3	B2.1

HFI, high-fall injury; PTAI, pedestrian traffic accident injury; PCAI, passenger car accident injury; BP, blood pressure; ER, emergency room; HR, heart rate; GCS, Glasgow Coma Scale; ISS, injury severity score; AIS, Abbreviated Injury Scale; AO-OTA, AO foundation and orthopedic trauma association.

### Initial presentation and diagnostic findings

Upon admission, all patients exhibited clinical signs of hemodynamic instability, accompanied by other manifestations of mild to moderate hemorrhagic shock. The admission systolic blood pressure ranged from 75 to 110 mmHg. The time from admission to intervention varied widely, ranging from 30 to 390 min, with only three patients undergoing embolization within one hour. One critically ill patient (Patient C) required REBOA as a bridge to definitive hemorrhage control. The median 24-h packed red blood cell (PRBC) transfusion for the six survivors was 4 units, while the single non-survivor (Patient D), who died from refractory shock, received a massive 28.5 units.

Six patients underwent CECT scans, and one patient underwent a non-contrast CT scan at the local hospital. This initial imaging identified active contrast extravasation in four patients (66.7%). Notably, in the remaining two patients (B and G), the CECT scan was negative for active bleeding. Despite these negative findings, the clinical team proceeded to angiography due to persistent hemodynamic instability and high-risk fracture patterns. Subsequent diagnostic angiography proved to be the definitive diagnostic tool, confirming active arterial hemorrhage in all seven patients (100%) (Table 2).

**Table 2:** Interventional strategies and outcomes in patients with hemodynamically unstable pelvic fracture.

Patient	CT findings	DTIT	Angio confirmed bleeding	Embolization technique	Embolic agent	Laterality	AE duration (min)	Initial pelvic fixation	Definitive pelvic fixation	24 h pRBC (units)
A	Extravasation	190 min	Yes	NSE	GF	Bilateral	75	None	None	8
B	No Extravasation	120 min	Yes	SE	GF	Unilateral	90	External (D1)	Iliolumbar (D8)	4.5
C^†^	Large pelvic hematoma	30 min	Yes	NSE	Coil	Bilateral	60	External (D1)	SI Screw (D4); Ant. Plate (D14)	3.5
D*	Extravasation	390 min	Yes	SE	GF	Bilateral	130	N/A	N/A	28.5
E	Extravasation	60 min	Yes	NSE	GF + Coil	Bilateral	85	External (D1)	None	3
F	Extravasation	60 min	Yes	NSE	Coil	Bilateral	75	External (D1)	SI Screw (D4)	3
G	No Extravasation	150 min	Yes	NSE	GF + Coil	Bilateral	68	None	Ant. Plate (D12)	7.5

AE, arterial embolization; REBOA, resuscitative endovascular balloon occlusion of the aorta; GF, gel foam; SI, sacroiliac; Ant., anterior; F/U, follow-up; NSE, non-selective embolization; SE, selective embolization; N/A, not applicable; pRBC, red blood cell; DTIT, door—to—intervention time; D, day.

† Non-contrast CT was performed for this patient.

* Patient died of hemorrhagic shock.

### Interventions and immediate outcomes

All seven patients underwent IIAE. The embolization techniques were tailored to the angiographic findings; a non-selective approach was used in five cases and a selective approach in two. Gel foam was the most common embolic agent, used either alone or in combination with coils in six patients. The embolization was performed bilaterally in six cases (Table 2).

The primary clinical outcome was highly successful, with six of the seven patients (85.7%) achieving immediate and sustained hemodynamic stability post-procedure. The one mortality (Patient D) occurred in a patient with an exceptionally high admission heart rate (178 bpm) and a prolonged embolization duration (130 min), who required a massive transfusion of 28.5 units of PRBCs and ultimately died to refractory hemorrhagic shock (Tables 1, 2). For the six survivors, pelvic external fixation was applied to four patients for initial stabilization, followed by staged internal fixation.

### Complications and follow-up

The in-hospital morbidity for survivors was low, with one patient developing pneumonia and another a deep vein thrombosis (DVT), both of which were managed successfully (Table 3). For the surviving patients, the median length of stay in the Intensive Care Unit (ICU) was 4 days, and the median total hospital stay was 30 days.

**Table 3:** Postoperative complications and recovery metrics*.

Patient	Incision/pin infection	Pneumonia	DVT	Gluteal muscle necrosis	Independent walking at F/U	Fracture healing at F/U	ICU LOS (days)	Hospital LOS (days)	Follow-up duration (months)
A	None	Yes	None	None	Yes	Yes	20	131	30
B	None	None	None	None	Yes	Yes	3	18	22
C	None	None	None	None	Yes	Yes	7	25	22
D	N/A	N/A	N/A	N/A	N/A	N/A	1	1	N/A
E	None	None	Yes	None	Yes	Yes	0	30	20
F	None	None	None	None	Yes	Yes	5	37	26
G	None	None	Yes	None	Yes	Yes	4	60	12

DVT, deep vein thrombosis; ICU, Intensive Care Unit; LOS, length of stay; N/A, not applicable; F/U, follow-up.

All six survivors were followed for a median of 22 months. At the final follow-up, the functional outcomes were excellent. All survivors had achieved solid fracture healing and were able to walk independently. Crucially, no long-term complications directly attributable to embolization, such as gluteal muscle necrosis or limb ischemia, were reported in any of the survivors (Table 3).

## Discussion

This multicenter case series, one of the few from China to focus on this topic, provides critical insights into the management of HUPF in children and adolescents. Our primary findings underscore that IIAE is a highly effective, life-saving intervention with an excellent safety profile in this vulnerable population. It is important to interpret these findings, however, through the lens of a retrospective study design that inherently does not capture eligible patients who did not survive to undergo the procedure. Three noteworthy observations from our study warrant detailed discussion: (1) the diagnostic discrepancy between CECT and angiography (33.3% CECT miss rate), (2) the paramount importance of a proactive treatment strategy tailored to pediatric physiology, and (3) the ongoing debate regarding selective vs. non-selective embolization as a damage control tactic.

Before delving into our findings, it is crucial to clarify the rationale for focusing on patients aged 15 and under. This study was intentionally designed to address two critical issues. First, this skeletally immature population is fundamentally different from adults; the period prior to the closure of the triradiate cartilage involves unique pelvic anatomy, biomechanics, and physiological responses to trauma (2). Therefore, the efficacy and safety of IIAE in this specific demographic cannot be simply extrapolated from adult data. Second, this pediatric and early adolescent group often represents an overlooked patient population, sometimes falling into a treatment gap between pediatric-focused institutions and adult trauma systems (8, 9). Our study aimed to provide targeted evidence for this distinct and clinically challenging group, justifying the focused cohort to ensure the clarity and specificity of our conclusions.

A pivotal finding of our study was the limited sensitivity of CECT in this clinical scenario. While CECT is an essential tool in initial trauma evaluation, it failed to detect active arterial bleeding in two of our six patients who underwent CECT, both of whom were later confirmed to have significant hemorrhage on angiography (Box 1). This observation aligns with research in adult populations, such as Nakajima's study, which documented a cohort of 47 patients where angiography detected bleeding missed by CECT, consistent with our findings (10). These results strongly suggest that for a pediatric patient with a high-risk pelvic fracture and persistent hemodynamic instability, a negative CECT should not preclude or delay angiography. The decision to proceed to interventional radiology should be driven by the patient's physiological state, not solely by initial imaging results (Figure 1). This principle is further supported by Katayama et al. (11), who demonstrated the value of proactive angiography in their study of 1,351 patients with pelvic fracture aged 19 and under. Among these patients, 221 (16.4%) underwent pelvic angiography, and propensity score matching revealed significantly lower in-hospital mortality rates in the angiography group compared to those who did not receive the procedure.

Successful hemorrhage control was achieved in six of seven patients (83.3%) in our cohort, reinforcing the efficacy of IIAE. This outcome is comparable to or better than those reported in other pediatric series, such as the 95% effective bleeding control rate in Vo's study (12) and the successful hemostasis in three of four patients reported by Gonzalez et al. (13). We attribute this success to a proactive treatment philosophy. While some literature expresses caution regarding early intervention in children (13), our findings advocate for a different approach. As ATLS guidelines emphasize, children possess remarkable compensatory mechanisms but can decompensate with catastrophic speed once these reserves are exhausted (6). Relying on blood pressure as the sole indicator of shock is unreliable and dangerous in this population. Therefore, the presence of compensated shock (e.g., persistent tachycardia despite resuscitation) in the setting of a severe pelvic fracture should be a clear indication for immediate, life-saving intervention.

A critical observation from our data is the significant variability in treatment timelines, which warrants further discussion. The prolonged pre-hospital times for transferred patients, ranging from 420 to 480 min, highlight a systemic challenge. In the context of a growing trend toward centralizing severe pediatric trauma care in China, our findings underscore the importance of proactive, timely transfer rather than delayed transfer compelled by clinical deterioration. These extended pre-hospital periods represent critical windows of ongoing hemorrhage and deepening shock that can adversely affect outcomes.

Furthermore, the wide variation in admission-to-intervention times, from 30 to 390 min, suggests a lack of standardized protocols for activating early angioembolization across different trauma centers. While some institutions demonstrated high efficiency, with three patients undergoing embolization in ≤1 h, significant delays in others may indicate that the vital role of early embolization is not yet universally prioritized. This is particularly concerning, as existing research has demonstrated that delayed embolization is associated with increased mortality in patients with pelvic fractures. Therefore, reducing these pre-hospital and in-hospital time intervals represents a crucial target for future quality improvement initiatives in the management of pediatric HUPF.

Our findings also contribute to the long-standing debate on the primary source of bleeding in HUPF. The historical belief, often misattributed to Huittinen and Slätis's cadaveric study (14), that venous bleeding predominates (85%) (15, 16) is challenged by our results. As has been correctly pointed out in the literature, that study could not confirm venous bleeding and actually showed widespread arterial extravasation (17). Our finding that 100% of our patients had arterial bleeding confirmed on angiography aligns with modern clinical research indicating a high prevalence (47%–97%) of arterial injury in HUPF (18–20). This reinforces the message that arterial hemorrhage is a primary driver of instability, and clinicians must maintain a high index of suspicion for internal iliac artery system injuries.

The debate between selective and non-selective embolization techniques is particularly relevant in damage control surgery. In our series, the sole mortality occurred in a patient who underwent a prolonged (130-min) selective embolization. While our sample size prevents a direct comparison, this observation aligns with concerns that attempting to selectively embolize multiple, scattered bleeding sites can significantly prolong procedure time, thereby extending the duration of shock and exacerbating coagulopathy (21–23). In contrast, non-selective embolization of the main internal iliac artery trunks is technically simpler, faster, and may be a more appropriate damage control strategy, a concept supported by others in the literature (21, 24). While Hymel et al.'s study in adults found no difference in procedure time (25), the unique context of pediatric trauma resuscitation may place a higher premium on speed.

Finally, the safety of IIAE is a critical consideration. Although the literature documents potential ischemic complications such as gluteal necrosis (26, 27), bladder necrosis (28), and fracture non-union (29) are well-documented. It is therefore highly reassuring that in our cohort, with a median follow-up of 22 months, no such long-term, procedure-related complications were observed. Of particular concern in children and adolescents is the preservation of future reproductive and sexual function. Reassuringly, the two male survivors in our cohort had documented normal erectile function during their hospitalization. This observation aligns with existing literature, which suggests that male sexual dysfunction is more often attributable to the severe pelvic fracture itself, including associated nerve damage, than to the embolization procedure (30, 31). Similarly, there is currently no evidence to suggest that bilateral internal iliac artery occlusion adversely affects female fertility, though continued long-term follow-up for the female adolescents in our study is warranted (32). Furthermore, all survivors achieved fracture healing and returned to normal daily activities, demonstrating that the robust collateral circulation in children may offer protection against the ischemic risks seen in adults.

### Limitations

This study has several limitations. The primary limitation is the small sample size, which is a direct consequence of our intentional focus on the rare clinical entity of HUPF within a specific, skeletally immature age group. While this approach provides targeted insights, it precludes formal statistical analysis and limits the generalizability of our conclusions. Second, the retrospective design introduces potential biases. An inherent selection bias exists because our cohort was identified based on patients who had already received IIAE. This methodology excludes eligible patients who did not undergo the procedure (e.g., due to early mortality), which may lead to an overestimation of its effectiveness. Furthermore, the data is subject to information bias related to the quality and completeness of medical records. Finally, the multicenter nature may introduce minor variations in treatment protocols.

## Conclusion

Our case series provides important evidence from China supporting the prompt and proactive use of IIAE in skeletally immature children and early adolescents with HUPF. IIAE is a safe and effective method for achieving rapid hemorrhage control with excellent long-term functional outcomes and no major observed ischemic complications. We strongly recommend that clinical teams maintain a low threshold for proceeding to angiography, even in the face of a negative CECT scan, and consider non-selective embolization as a primary damage control technique in these critically injured children. Future large-scale, prospective studies are warranted to establish definitive management protocols by validating the clinical decision pathway for angiography despite negative CT findings, focusing on minimizing time-to-intervention, and incorporating systematic long-term functional follow-up.

Box 1 Illustrative case summaries.

**Illustrative Case 1: Patient B**

A 12-year-old female presented with post-transfusion hypotension (75/45 mmHg) 7 h after a fall. Although CECT confirmed a severe sacroiliac joint dislocation, it showed no active arterial extravasation. However, due to the high-risk fracture, a large retroperitoneal hematoma, and the initial shock state, angiography was performed. The procedure confirmed and controlled active bleeding from the left obturator artery via selective embolization. The patient was stabilized, underwent definitive pelvic fixation 8 days later, and was discharged with a good recovery.

**Illustrative Case 2: Patient C**

A 12-year-old male presented 30 min after a fall with unstable hypotension (93/55 mmHg) necessitating REBOA. A CT scan revealed a large retroperitoneal hematoma. Subsequent angiography identified bleeding from the right iliolumbar artery and other scattered sites, which were controlled with a combination of gelatin sponge and coil embolization. The patient was stabilized and underwent staged fixation for his multiple fractures. After a 7-day ICU stay, he was discharged on day 25.

## Data Availability

The original contributions presented in the study are included in the article/Supplementary Material, further inquiries can be directed to the corresponding author.
