# Correction: Angioembolization as a lifesaving maneuver for unstable pelvic fractures in skeletally immature children: a multicenter case series

**DOI:** 10.3389/fped.2026.1783739

**Published:** 2026-01-22

**Authors:** Frontiers Production Office

**Affiliations:** Frontiers Media SA, Lausanne, Switzerland

**Keywords:** children, adolescent, pelvic fracture, internal iliac artery, angiography, embolization, hemorrhagic shock

**Author affiliation**

The affiliation list in the published article was not correct. The corrected list of affiliations is below.

1. Department of Traumatology, Chongqing University Central Hospital, Chongqing Emergency Medical Center, Chongqing, China

2. Department of Cardiothoracic Surgery, The 909th Hospital, School of Medicine, Xiamen University, Zhangzhou, China

3. Department of Emergency, The Second Affiliated Hospital, Hengyang Medical School, University of South China, Hengyang, China

4. Trauma Center, Pediatric Orthopedic Department, The Second Affiliated Hospital, Hengyang Medical School, University of South China, Hengyang, China

5. Department of Emergency, Affiliated Hospital of Zunyi Medical University, Zunyi, China

6. Emergency Surgery Department, Shaanxi Provincial People's Hospital, Xi'an, China

7. Department of Emergency Medicine, Second Affiliated Hospital, Zhejiang University School of Medicine, Hangzhou, Zhejiang, China

**Error in Author List**

The order of authors in the author list of the published paper was erroneously given as:

Mao Zhang^1^*, Dingyuan Du^2^*, Gongliang Du^3^, Anyong Yu^4^, Yong Fu^5^, Yong Luo^6^, Junhua Guo^7^, Yunfeng Yi^7^, Guangbin Huang^2^ and Hui Li^1†^

The correct author list reads:

Hui Li^1^, Guangbin Huang^1^, Yunfeng Yi^2^, Junhua Guo^2^, Yong Luo^3^, Yong Fu^4^, Anyong Yu^5^, Gongliang Du^6^, Mao Zhang^7^, and Dingyuan Du^1*^

The original version of this article has been updated.

